# Construction of a novel rabbit model of ureteral calculi implanted with flowable resin

**DOI:** 10.1186/s12894-022-01056-x

**Published:** 2022-07-18

**Authors:** Hao Su, Heng Liu, Ke Yang, Weiming Chen, Dongbo Yuan, Wei Wang, Guohua Zhu, Bin Hu, Kehua Jiang, Jianguo Zhu

**Affiliations:** 1 Department of Urology, Guizhou Provincial People’s Hospital, Guiyang, 550002 Guizhou Province China; 2 Zunyi Medical University, Zunyi, 563000 Guizhou Province China; 3 Guizhou Medical University, Guiyang, 550002 Guizhou Province China; 4 Guizhou University School of Medicine, Guiyang, 550025 Guizhou Province China

**Keywords:** Urinary calculi, Experimental model, Flowable resin, Rabbit

## Abstract

**Background:**

The purpose of this study was to characterize the pathophysiological changes of hydronephrosis caused by ureteral calculi obstruction in a new rabbit ureteral calculi model by implanting flowable resin.

**Methods:**

Forty New Zealand rabbits were randomly divided into two groups: the calculi group and the sham control group. In the calculi group (n = 20), rabbits were operated at left lower abdomen and the left ureter was exposed. Then flowable resin (flowable restorative dental materials) was injected into the left ureter using a 0.45 mm diameter intravenous infusion needle. Then light-cured for 40 s by means of a dental curing light to form calculi. In the sham control group, normal saline was injected into the ureter. Rabbits underwent X-ray and routine blood and urine tests preoperatively, as well as X-ray, CT, dissection, HE staining and routine blood and urine tests on 1, 3, 5 and 7 days postoperatively. Stone formation was assessed by X-ray and unenhanced CT scan after surgery. The pathophysiological changes were evaluated through dissection, HE staining and routine blood and urine tests.

**Results:**

Ureteral calculi models were successfully constructed in 17 rabbits. In calculi group, high-density shadows were observed in the left lower abdomen on postoperative day 1st, 3rd, 5th and 7th by X-ray and CT scan. Dissection found obstruction formation of the left ureters, dilatation of the renal pelvis and upper ureter during 7 days after surgery. The renal long-diameters of the left ureters increased only on the 1st postoperative day. HE staining found ureteral and kidney damage after surgery. In calculi group and sham group,the serum creatinine, urea nitrogen, white blood cells and urine red blood cells were raised at day 1 after surgery. However, the indicators returned to normal at day 3, 5, and 7.

**Conclusions:**

This is a stable, less complicated operation and cost-effective ureteral calculi model by implanting flowable resin. And this novel model may allow us to further understand the pathophysiology changes caused by ureteral calculi obstruction.

## Background

Ureteral calculus is one of the most common diseases of urology [1]. Due to the effects of the increasing incidence of obesity, diabetes, and changes in dietary habits, the morbidity and recurrence rates of ureteral calculi are gradually rising [2, 3]. When the stone moves into the ureter, it will block the ureter and induce ureteral spasm [4]. Ureteric muscle spasm can cause a major symptom of ureteric calculi: renal colic. If the obstruction is not relieved, it will cause a range of serious complications such as reduced renal function, Hydronephrosis, urinary tract infection, ureteral stricture, etc. These changes ultimately contribute to kidney failure and seriously threaten the life safety of patients [5].

At present, the pathophysiological mechanisms of ureteral calculus remain incompletely defined. Therefore, we need animal models of ureteral calculi to study. There are relatively few reports on models of ureteral calculi. Soria et al. constructed ureteral stone models that were created surgically introducing artificial ureteral stones in both renal pelvises of pigs [6, 7]. But the price is higher and the operation is complex. The primary role of this model is to perform training in retrograde intrarenal surgical skills. In the present study, we established a novel rabbit model of ureteral calculi with simple manipulation, reasonable price and stable by implanting flowable resin. It enables us to better study the pathophysiological changes of ureteral calculi.

## Materials and methods

### Experimental animals

We used Forty New Zealand rabbits of either sex, (4 ± 0.5) months of age and weighing (3.0 ± 0.5) kg, provided by the Laboratory Animal Center of Guizhou Medical University, with the license number: SCXK-2018-001. Rabbits were housed in individual cages at a temperature between 17 to 19 °C with humidity of 40–60% on a 12-h light/dark cycle and fed a normal chow diet. The animal model research was reviewed and approved by the Medical Ethics Committee of Guizhou Provincial People's Hospital (Ethical Approval No.: 2017059). All experimental procedures were conducted in accordance with the 1996 revision of the Guide for the Care and Use of Laboratory Animals issued by the National Research Council Laboratory Animal Resources Institute.

### Preoperative treatment

**Fig. 1:**
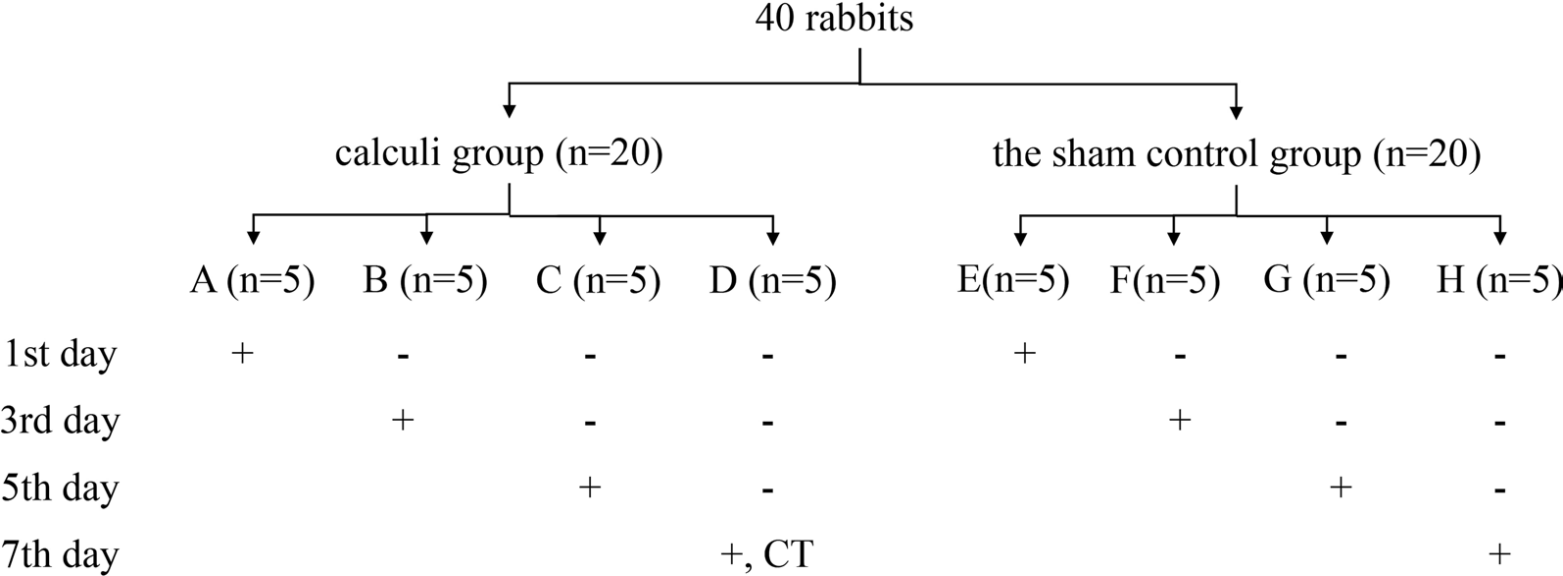
Experimental flow chart. +: X-ray, dissection, collection of blood and urine. −: No treatment

Forty rabbits were randomly divided into the calculi group and the sham control group, 20 rabbits in each group (Fig. 1). Before surgery, when the rabbits in calculi and sham control group were anesthetized, the chest X-ray examinations in prone position were performed on the lower abdomen by a small animal DR machine. Then five from all rabbits were randomly selected to obtain blood from the vein of rabbit ear margin and urine collected by bladder massage by bladder massage.

### Experimental procedures

All rabbits were fasted before surgery but allowed to drink water overnight. After being weighed, each rabbit was anesthetized by intravenous injection of sodium pentobarbital (30 mg/kg, Sinopharm Chemical Reagent Co., Ltd., Shanghai, China). The rabbit was placed on the operating table in the supine position. Skin in the left lower abdomen was clipped and disinfected with iodophor. The surgical area was draped with a sterile surgical towel. A 5 cm incision was made in the left lower abdomen and the subcutaneous tissue, muscle, and peritoneum were incised. The colon and small intestine were mobilized to expose the left kidney and ureter. We selected the ureter at a distance of 4 cm from ureteropelvic junction and gently lifted 1 cm ureter with hemostatic forceps. The intravenous infusion needle with a diameter of 0.45 mm (needle tip towards the kidney) was inserted into the ureteric lumen.

**Fig. 2:**
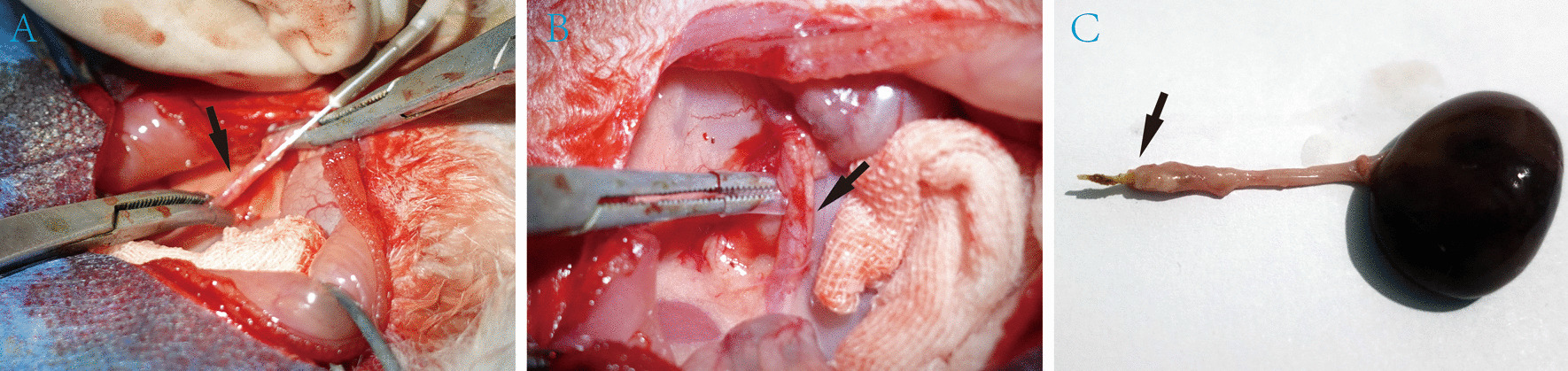
**A** Flowable resin was injected with a 0.45 mm diameter intravenous infusion needle. **B** Flowable resin in the ureter was light-cured for 40 s by a dental curing light. **C** Flowable resin was present in ureter lumen by anatomic dissection. The stone location was marked by black arrows

In the calculi group, we injected 0.1 ml flowable resin (FiltekTM Z350 XT Flowable Restorative) (Fig. 2A). Care was taken throughout to minimize damage to ureter. Flowable resin in the ureter was light-cured for 40 s by means of a dental curing light (R&D, IvoclarVivadent AG, Schaan, Principality of Liechtenstein, 2013) operated at 550 mW/cm^2^ and subsequently hardened to form stones (Fig. 2B). In the sham control group, we injected with the same volume of saline. Subsequently, the same dental curing light was exposed for 40 s.We took photographs and recorded the position of the stones. After ensuring no incidence of urinary leakage, bowel was repositioned and the abdomen was closed with 3–0 resorbable sutures. Then, the X-ray examinations were again taken. All operations were performed by the two senior urological Surgeons. Prophylactic antibiotic(brizolina) was given preoperatively and intramuscular injections of brizolina were given for 3 days after surgery.

### Postoperative treatment

After surgery, The calculi group was randomly divided into 1st day (A), 3rd day (B), 5th day (C), 7th day (D) postoperatively according to the number of postoperative days. Similarly, the sham control group was randomly divided into 1st day (E), 3rd day (F), 5th day (G), 7th day (H) postoperatively. Each group has 5 rabbits. The schematic diagram of experimental groupings is shown in Fig. 1. At 1 day after surgery, rabbits in group A and E were anesthetized by intravenous injection of sodium pentobarbital. In group A, X-ray examinations were conducted in rabbits of the lower abdomen. We observed whether stone locations had changed by comparing immediate postoperative X-ray with the 1st day postoperative X-ray of the same rabbit.

Subsequently, rabbits in group A and E were euthanized by overdose of intravenous pentobarbital sodium. Kidneys and ureters were dissected and exposed through the median abdominal incision. Then they were photographed and recorded. Blood and urine samples from all rabbits were collected. Kidneys and ureters were removed from the rabbits. We measured the long-diameter of the kidneys bilaterally and renal pelvic width. Rabbits in groups B, F on the 3rd postoperative day, groups C, G on the 5th postoperative day, and groups D, H on the 7th postoperative day were operated similarly to groups A, E, respectively. On the 7th postoperative day, high-resolution computed tomography (CT) was performed on two randomly chosen rabbits in group D to observe the locations of calculi from different angles (axial, coronal, and sagittal).

### Histopathology assessment

The bilateral kidneys and ureters were fixed in 4% paraformaldehyde, dehydrated with alcohol, embedded in paraffin, sectioned, stained with hematoxylin and eosin. Pathological changes were observed under a microscope. The extent of renal tubule damage was recorded using a renal histopathological scoring system as follows: 0, normal; 1, areas of tubular epithelial cell swelling, vacuolar degeneration or necrosis involving < 25% of cortical tubules; 2, similar changes involving > 25% but < 50% of cortical tubules; 3, similar changes involving > 50% but < 75% of cortical tubules; and 4, similar changes involving > 75% of cortical tubules. The mean score of each successive field (magnification × 200) was taken as the renal histopathological score of that kidney section.

### Blood and urine routine examination

Blood parameters including white blood cell (WBC), blood urea nitrogen (BUN) and serum creatinine (Scr). Urine parameters including urine red blood cell (URBC) and urine white blood cell (UWBC).

### Statistical analysis

All data were expressed as mean ± standard deviation. The statistical analysis of the experimental results was conducted with the software SPSS 19.0 (SPSS Inc., Chicago, IL, USA), and presented with GraphPad Prism 5.0 (GraphPad Software, Inc., La Jolla, CA, United States). The difference between groups was statistically significant (*P* < 0.05).

## Results

### The general condition of the rabbits

The operation time per animal did not exceed 20 min. After surgery, all rabbits were fed separately under the same conditions, Unfortunately, one rabbit in group B died on the 1st postoperative day due to intolerance to surgery. Therefore, it was excluded from the study. The rest of the rabbits completed the study protocol.

### Imaging examinations

**Fig. 3:**
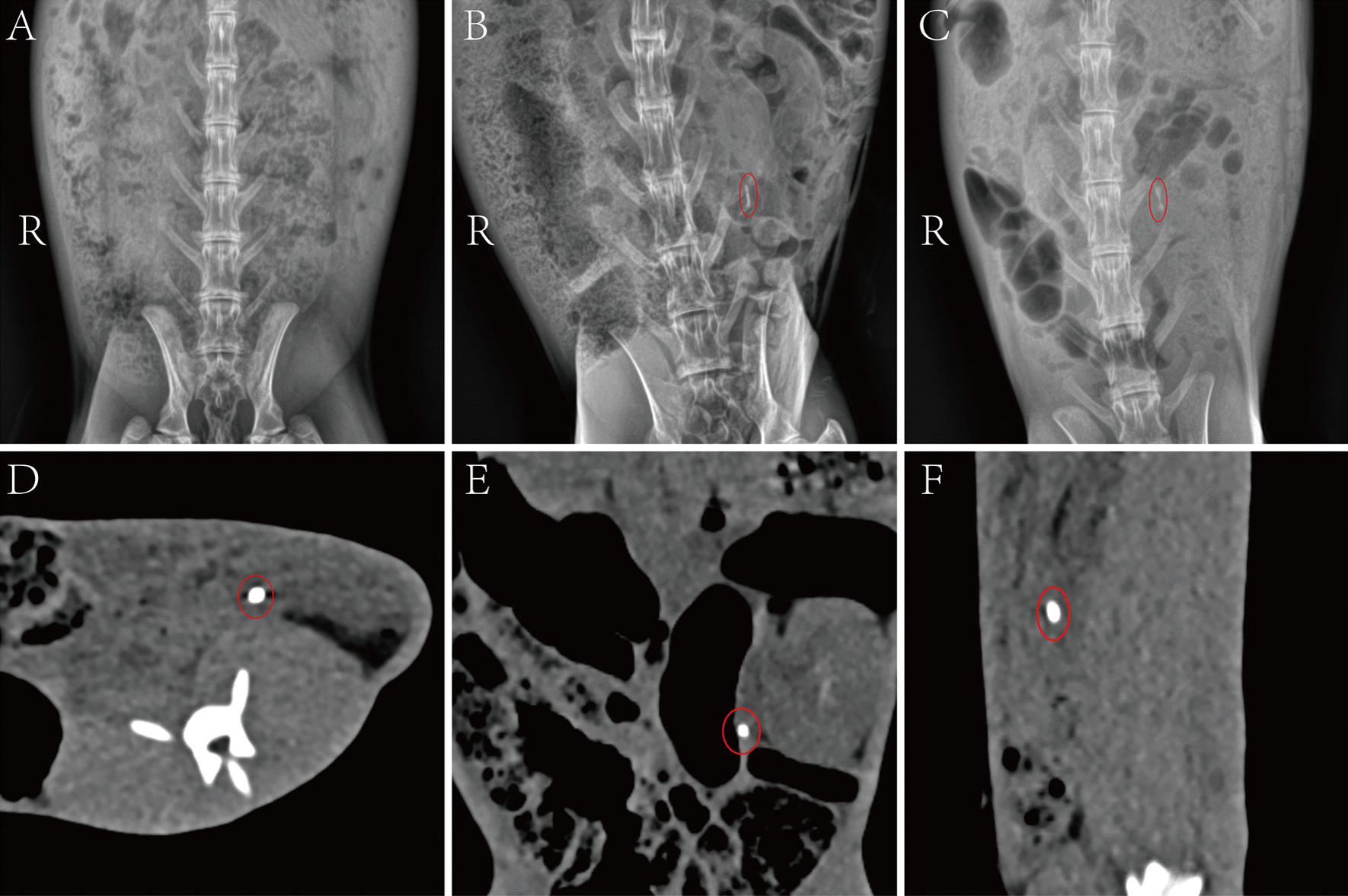
Ureteral stones were assessed by X-ray and CT imaging. **A** The preoperative x-ray. **B** The end of the surgery X-ray. **C** The 7th postoperative day X-ray. The 7th day postoperatively CT examination of cross-sectional (**D**), Coronal (**E**) and sagittal scans (**F**). The high-density shadows of stones were marked by red circles

X-ray examination showed there was no significant abnormality in the lower abdomen before surgery (Fig. 3A). In the calculi group, there was a high-density shadow in the lower left abdomen of each rabbit at the end of surgery (Fig. 3B). Rabbits in groups A, B, C and D, respectively, also had a high-density shadow at 1, 3, 5 and 7 days after surgery (Fig. 3C). Stone location of each rabbit has no significant changes by comparing two postoperative imaging results. In the sham control group, there were no differences between the preoperative and the end of surgery in X-rays.

CT scan showed that high-density shadows were found in the left ureteral distribution area with cross-sectional, coronal, and sagittal scans (Fig. 3D, E, F). The stone density were ranged from 400 to 1200 HU.

### Dissection analysis

**Fig. 4:**
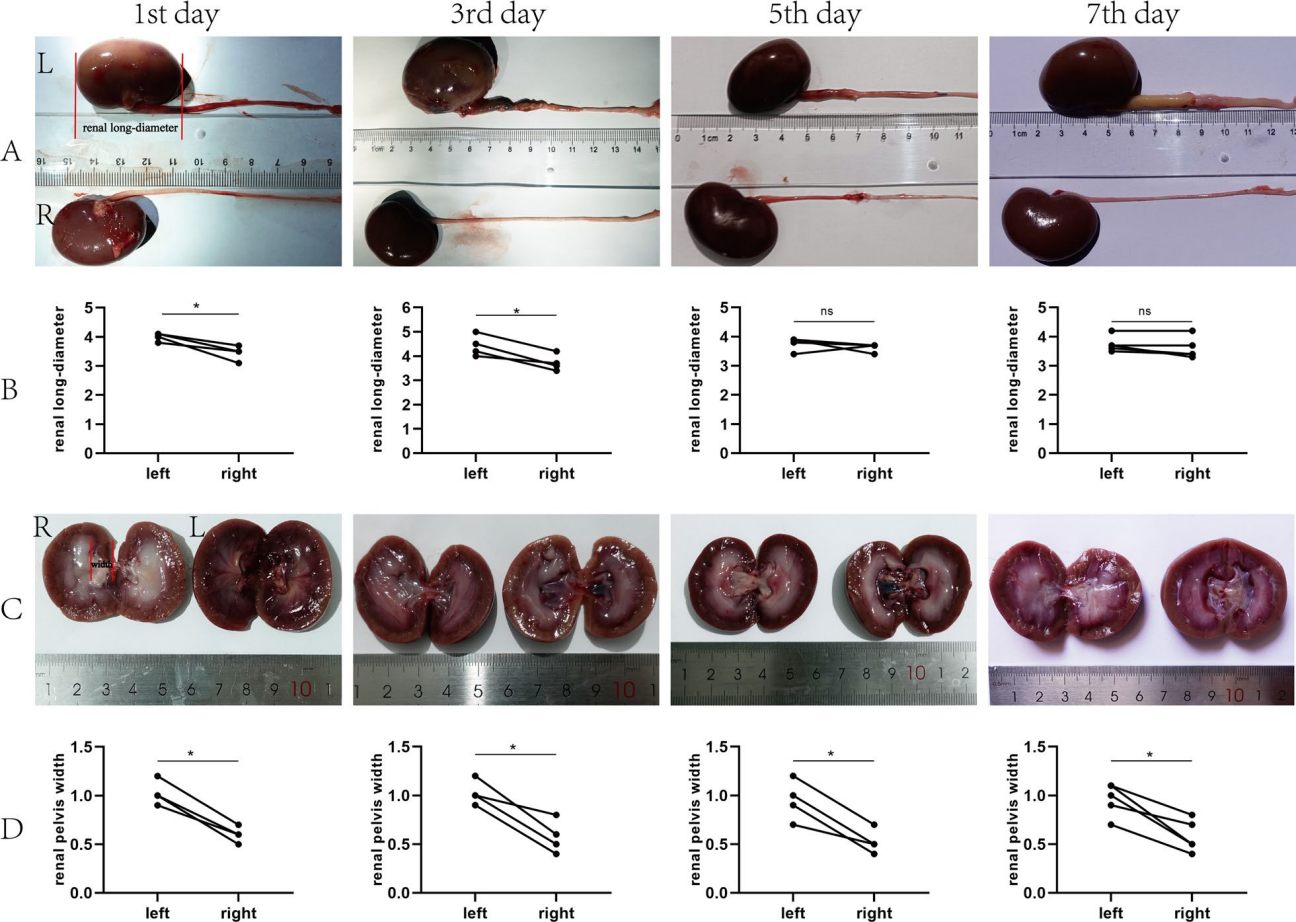
Pictures of kidneys and ureters in the calculi group. **A** Bilateral kidneys and ureters. **B** Comparison of bilateral renal long-diameters. **C** Kidney longitudinal sections. **D** Comparison of bilateral renal pelvis width. *P < 0.05

**Fig. 5:**
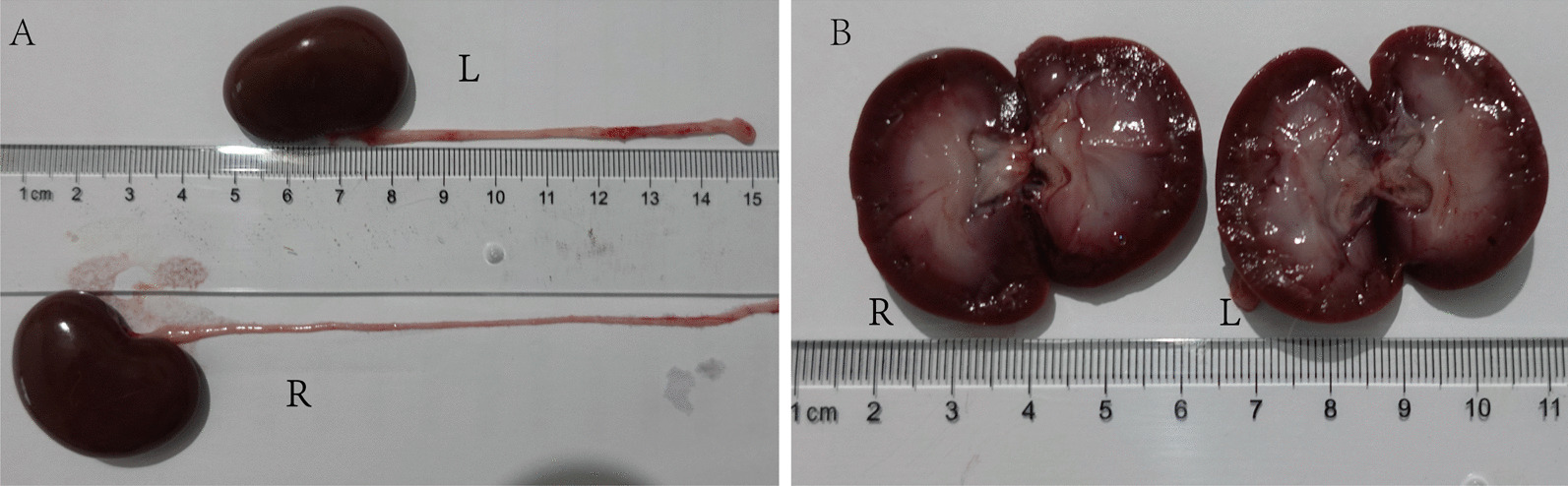
Pictures of kidneys and ureters in sham control group on the 3rd postoperative day. The renal long-diameters (**A**) and renal pelvis width (**B**) were no significant difference

On the 1st and 5th day postoperative day, one rabbit in group A and C, respectively, was dissected to found the calculi were not in the ureteral lumen, but in the mucosal layer. Therefore, they were excluded from the experiment. In the calculi group, rabbits were euthanized at each time point of 1, 3, 5, and 7 days after the procedure respectively. Flowable resin (calculi) was present in ureter lumen by anatomic dissection (Fig. 2C). They were located to create the obstruction in the ureter lumens. We could observe inflammatory adhesions of the periureteral tissues where the calculi were located on the left side, and upper ureters dilations with calculi. The long diameters of the left kidneys were different on the 1st and 3rd postoperative days compared with contralateral kidneys (*P* < 0.05) (Fig. 4A, B). However, we found that the diameter of the left renal pelvis was larger than that of the right kidneys by longitudinal axis (*P* < 0.05) (Fig. 4C, D). In the sham control group, long diameters and renal pelvis width of bilateral renal were not different postoperatively (Fig. 5).

### Histopathological analysis

**Fig. 6:**
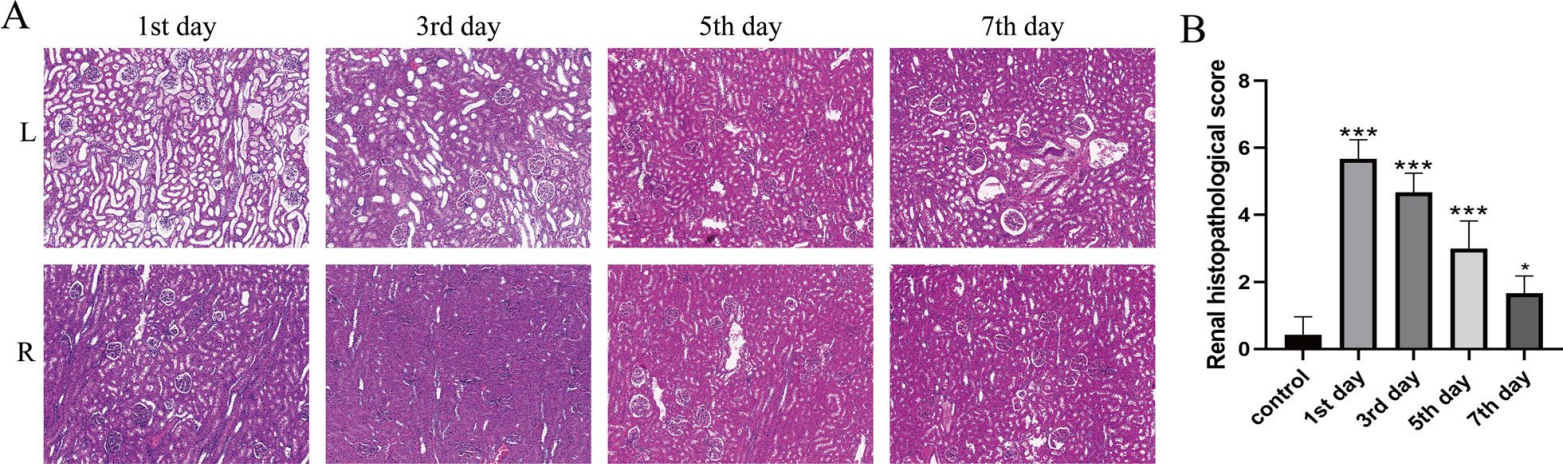
Pathological changes of kidneys. **A** The left kidneys had varying degrees of pathological damages. The right kidney had no significant changes. H&E stain 10X. **B** Renal histopathological score. ***: *P* < 0.001 vs. control group. *: *P* > 0.05 vs. control group

**Fig. 7:**
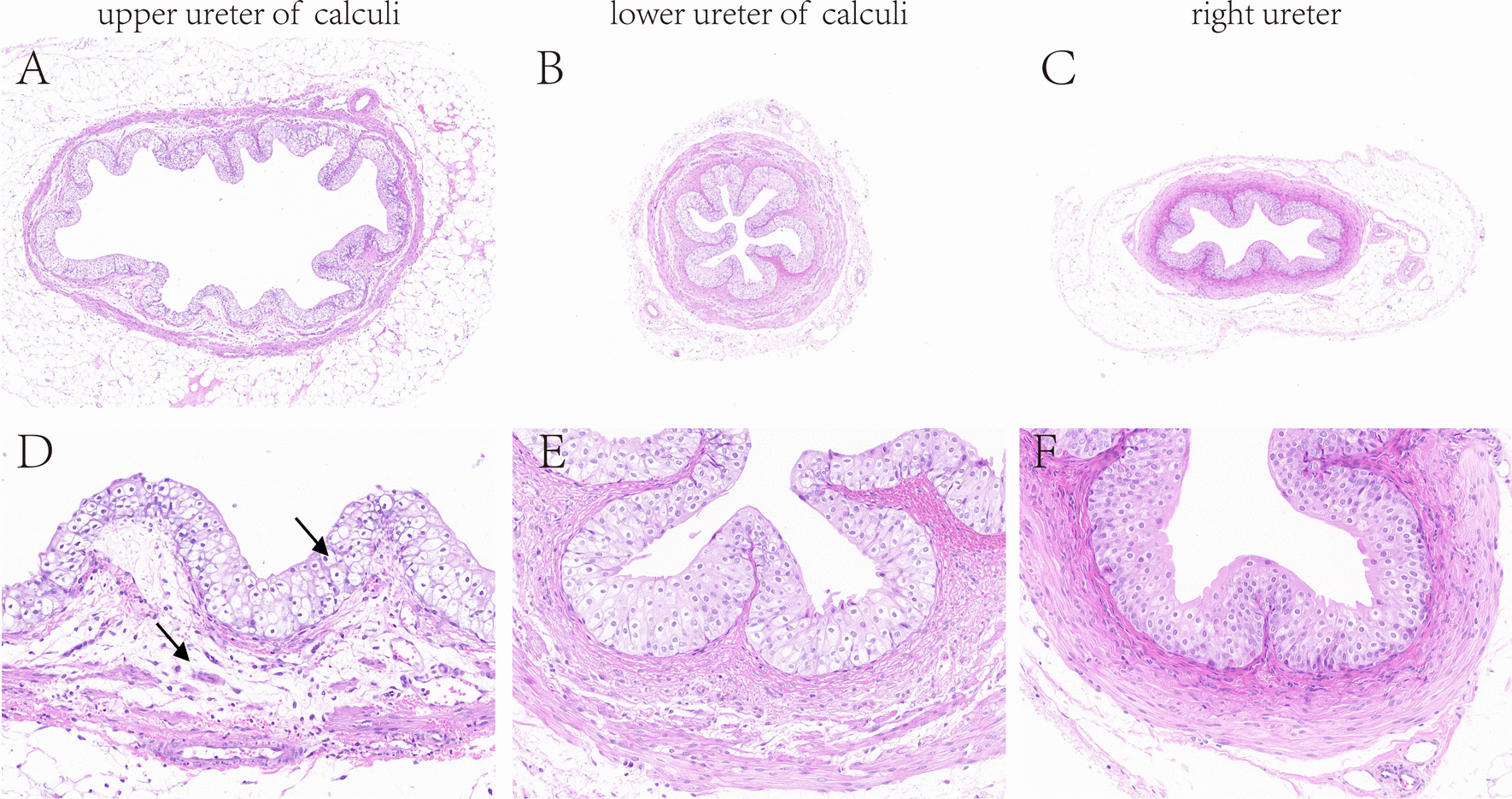
Pathological changes of ureters. The upper ureter of calculi was dilation. Endothelial cells were disorderly arranged and connective tissues were edema. Lower ureter of calculi and right ureter are normal. **A**, **B**, **C**: H&E stain 5X. **D**, **E**, **F**: H&E stain 20X

In the calculi group, we could observe various degrees of tubular lesions postoperatively in the left kidneys. But no significant changes were observed in right kidneys (Fig. 6A). We used the right kidneys as control group. Then, the kidney pathology score was used for evaluating the severity of the renal injury. The results showed that kidney damages were most severe on the 1st postoperative day, and the damages would gradually decrease with time (Fig. 6B). In the sham control group, there was no significant difference in renal histopathological scores among the groups. We divided the ureter into upper ureter of calculi, lower ureter of calculi and right ureter. By comparison at 5 × magnification, upper ureter of calculi was dilation. Some epithelial cells were deficiency and disarranged. The connective tissues were edema. Muscular layer tissues showed disorganized structure. However, histological structure in lower ureter of calculi and right ureter were normal (Fig. 7). Likewise, histological structure of ureters also was normal in the sham control group.

### Blood and urine routine

We set the preoperative blood and urine routine as group N. In the calculi group, serum creatinine, blood urea nitrogen, white blood cells, and urine red blood cells were increased on the 1st postoperative day. And they were recovered on the 3rd, 5th and 7th days. There was no significant change in urine white blood cell. In the sham control group, the changes of blood and urine routine were similar to the calculi group (Table 1).

**Table 1:** Scr, BUN, WBC, URBC and UWB in experimental rabbits

Group	Scr μmol/L	BUN mmol/L	WBC X10^9^/L	URBC pcs/μL	UWBC pcs/μL
N	63.2 ± 7.4	8.1 ± 1.2	5.6 ± 1.6	1.5 ± 1.2	1.6 ± 1.2
A	83.0 ± 19.7	21.9 ± 1.9	13.6 ± 3.6	675.8 ± 307.7	2.2 ± 2.3
B	77.9 ± 13.1	8.3 ± 1.1	7.3 ± 2.4	4.0 ± 5.1	2.4 ± 2.6
C	66.2 ± 8.3	8.0 ± 1.4	6.5 ± 0.9	2.6 ± 3.3	2.8 ± 3.1
D	67.8 ± 6.8	6.8 ± 1.7	6.3 ± 2.8	1.4 ± 2.2	4.2 ± 4.2
E	85.0 ± 8.9	17.7 ± 1.6	14.7 ± 3.4	1124.4 ± 478.2	1.2 ± 1.3
F	73.8 ± 10.2	8.0 ± 1.2	7.6 ± 1.7	0.4 ± 0.9	1.6 ± 1.6
G	77.4 ± 19.1	8.8 ± 1.0	6.5 ± 1.4	1.2 ± 0.9	1.6 ± 2.6
H	73.7 ± 15.6	8.9 ± 2.8	6.2 ± 2.4	1.4 ± 1.8	3.8 ± 4.7

N: Preoperative. The calculi group: (A) 1st day, (B) 3rd day, (C) 5th day, (D) 7th day. The sham control group: (E) 1st day, (F) 3rd day, (G) 5th day, (H) 7th day. Values are presented as mean ± standard deviation

## Discussions

There are many methods for the construction of urinary stone models. The technically mature and widely used is the kidney stone model. For example, Calcium oxalate precursors method by feeding or injecting substances on the pathway of oxalate metabolism to increase oxalate in animals is the most common method for the formation of urinary calculi [8–10]. However, the methods for the construction of ureteral stone models are less reported. As a large animal, the pig is an ideal object for calculi implantation in vitro. By means of percutaneous renal surgery, Some people use rigid nephroscope to guide gypsum artificial stones into renal pelvis of pigs [11–13]. This model is widely used to train skills about urological endoscope, observe the occurrence of complications after extracorporeal shock wave lithotripsy (ESWL), the success rate of renal puncture under X-ray guidance and other surgical therapeutic effects [12–16]. It also been used to construct ureter calculi model of the proximal [17]. This method requires the kidneys of large animals, so it is not applicable to small animals. Similarly, ureter calculi model can be surgically constructed in pigs [6, 7]. However, due to its high cost and ethical problems, pigs are not considered to be the best choice for animal models. Giamberardino MA and his team in Italy used a syringe to inject dental cement into the ureter of rats to produce a painful ureteral stone model [18–20]. The most important use of this model was to study pain, and it did not investigate the ureter and urinary system. So far, this model has mainly been applied in pain. No other team has replicated the model.

In this study, we constructed a simple ureteral calculi model by innovatively implanting foreign matter of flowable resin in New Zealand rabbits. We selected New Zealand rabbits as experimental animals, which were docile, cheaper than pigs, easy to feed, and medium in size to clearly observe local pathological changes [21]. The material, flowable resin, is a good choice as calculi material due to its characteristics of rapid setting time, light-cured and lowviscosity [22]. When it solidifies, no exothermic generation, thus there is less damage to the ureter. Another particularly excellent advantage of this material is that it presents a high-density [23]. We can observe the stones by X-ray or CT.

The stones were found to be in a stable position by two postoperative X-rays. In the clinical, non-enhanced computerized tomography was performed for patients when X-ray plain films and ultrasound are negative or equivocal [24]. We selected two rabbits in group D for CT scans to better see the location of the stones. We found it interesting that the CT values of the flowable resin were similar to calcium stones [25].

In the calculi group, we found increases in serum creatinine, urea nitrogen, white blood cells, and urine red blood cells at 1 day after surgery. Therefore, we set a corresponding sham control group, and found that these indexes increased too. We speculated that the increase of these indexes might be related to surgery. For the HE stain of the kidneys, we could observe interstitial edema and tubular dilation. It is similar to the early renal changes in unilateral ureteral obstruction [26].

Unilateral ureteral obstruction model is achieved by irreversible ligation of the ureter [27]. However, this method causes a complete obstruction. We implanted the stones into the lumen of the ureter by injecting flowable resin. The tissue and blood vessels in the upper and lower segments of the stone were not completely blocked. Therefore, the model we constructed was the unilateral ureteral incomplete obstruction model.

At the same time, our model also has several shortcomings. Surgical manipulations have ureteral injuries, and have an effect on the normal physiological indexes of experimental animals. Then there was certain difficulty to control the dosage because the light-cured flow resin has poor mobility. In the urological experimental studies, rabbit’s urine composition and density are distinguished from humans, as translation of results may not be reliable in certain aspects [28].

In our study, a new method of constructing ureteral calculus obstruction model with high success rate was proposed. We provided detailed operation steps. At present, most of the research on ureteral calculi is based on clinical studies. Our model provides useful research material to study the molecular alterations in the local pathophysiology of the ureter after ureteral calculi. By studying the histopathological changes at different days after the occurrence of calculi, it is possible to provide a reference for the appropriate time to operate on ureteral calculi. The expulsion of ureteral calculi is still inconclusive. According to the basic understanding of the structure and innervation relationship of ureteral smooth muscle, the expulsion of stones is not only associated with the effect of gravity in urine flow and bladder negative pressure, but also with the ureteral peristalsis [29]. Therefore, our model provides an important animal model for further research on ureteral calculus expulsion. Furthermore, we are also flexible in our surgical approach and can choose different locations for model construction depending on the purpose of the study.

## Conclusion

In our research, we constructed a novel rabbit model of ureteral calculi by implanting flowable resin. It has the characteristics of stable, less complicated operation and cost-effective. We can use the model for basic research on ureteral calculi.

## Data Availability

The images and datasets used in the current study is available from the corresponding author on reasonable request.
